# Zebrafish heart regeneration: 15 years of discoveries

**DOI:** 10.1002/reg2.83

**Published:** 2017-09-28

**Authors:** Juan Manuel González‐Rosa, Caroline E. Burns, C. Geoffrey Burns

**Affiliations:** ^1^ Cardiovascular Research Center Massachusetts General Hospital Charlestown MA 02129 USA; ^2^ Harvard Medical School Boston MA 02115 USA; ^3^ Harvard Stem Cell Institute Cambridge MA 02138 USA

**Keywords:** cardiomyocyte proliferation, heart regeneration, myocardial infarction, zebrafish

## Abstract

Cardiovascular disease is the leading cause of death worldwide. Compared to other organs such as the liver, the adult human heart lacks the capacity to regenerate on a macroscopic scale after injury. As a result, myocardial infarctions are responsible for approximately half of all cardiovascular related deaths. In contrast, the zebrafish heart regenerates efficiently upon injury through robust myocardial proliferation. Therefore, deciphering the mechanisms that underlie the zebrafish heart's endogenous regenerative capacity represents an exciting avenue to identify novel therapeutic strategies for inducing regeneration of the human heart. This review provides a historical overview of adult zebrafish heart regeneration. We summarize 15 years of research, with a special focus on recent developments from this fascinating field. We discuss experimental findings that address fundamental questions of regeneration research. What is the origin of regenerated muscle? How is regeneration controlled from a genetic and molecular perspective? How do different cell types interact to achieve organ regeneration? Understanding natural models of heart regeneration will bring us closer to answering the ultimate question: how can we stimulate myocardial regeneration in humans?

## 1 WHY STUDY HEART REGENERATION?

Millions of people die worldwide each year from myocardial infarctions (MIs, or “heart attacks”), the irreversible loss of heart muscle cells caused by prolonged myocardial ischemia. In the USA, someone dies about every 90 sec from an MI (Mozaffarian et al., 2015), and the costs associated with managing heart disease, including MIs, exceed that of any other diagnostic group (Mozaffarian et al., 2015). Alarmingly, the World Health Organization has predicted that cardiovascular diseases will become an epidemic in the coming decades as the population ages (Mendis, Puska, Norrving, & World Health Organization, 2011).

**Figure 1:**
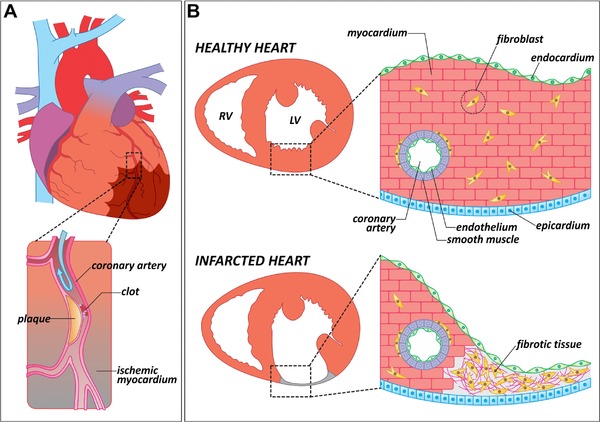
Causes and consequences of myocardial infarction in mammals. (A) Schematic representation of a human heart in which one of the coronary arteries is occluded by an atheromatous plaque (magnified area). When blood flow is interrupted, a region of the myocardium becomes ischemic (brown shade). Ischemic myocardium eventually dies and is replaced by fibrotic tissue. (B) Anatomical and histological differences between a healthy and an infarcted heart. In contrast to a healthy heart, the infarcted ventricle shows a thinning of the affected wall, in which the cardiac muscle has been replaced by fibrotic tissue. LV, left ventricle; RV, right ventricle

Most MIs are caused by the acute blockage of a coronary artery resulting from thrombus formation over an atheromatous plaque, a defining feature of atherosclerotic disease (Fig. 1A). In adult mammals, damaged muscle is irreversibly lost and replaced by a non‐contractile scar (Fig. 1B) (Pfeffer & Braunwald, 1990, reviewed in Frangogiannis, 2006). Fibrotic scarring maintains ventricular wall integrity but undermines pump function, often to the point of congestive heart failure (reviewed in Fuster, Walsh, & Harrington, 2011; Jessup & Brozena, 2003; Kehat & Molkentin, 2010). As a result, patients who experience an MI have a lower quality of life and often die prematurely. Despite medical advances in prevention and early intervention, MIs are currently incurable without heart transplantation, which is limited by organ donations. Otherwise, standard treatments are purely palliative (reviewed in Augoustides & Riha, 2009). The average life expectancy following an MI is lower than for most cancers (Stewart, MacIntyre, Hole, Capewell, & McMurray, 2001). Therefore, any therapies capable of stimulating myocardial regeneration would significantly reduce morbidity and mortality for millions of people every year.

In recent decades, scientists from several disciplines have worked hard to design therapeutic strategies for regenerating the human heart. As a result, significant advances have been made in the production of new biomaterials (reviewed in Coulombe, Bajpai, Andreadis, & Murry, 2014), the transplantation of stem cell derived cardiomyocytes (Chong et al., 2014; Kadota, Pabon, Reinecke, & Murry, 2017; Shiba et al., 2012), and the reprogramming of fibroblasts into new muscle (reviewed in Kojima & Ieda, 2017). As an attractive alternative to these approaches, scientists have also focused their attention on studying natural models of cardiac regeneration, such as certain amphibian and fish species. Here, we summarize historical and recent findings from the study of heart regeneration in adult zebrafish. We discuss significant advances in our understanding of the cellular and molecular mechanisms that govern cardiac regeneration and describe deficiencies in our knowledge as well as long term goals of the field.

## 2 HEART REGENERATION FROM A HISTORICAL PERSPECTIVE

We define regeneration as the structural and functional recovery of injured organs or lost body parts (reviewed in Poss, 2010). Mammals efficiently regenerate injuries to the liver, skeletal muscle, bones and, to some degree, skin. All of these organs recover their structure and function in the weeks or months following trauma. In contrast, the adult mammalian heart fails to recover structurally or functionally after injury.

Interestingly, the heart was not always considered a non‐regenerative organ. At the beginning of the 20th century, it was mostly accepted that the adult human myocardium had some ability to regenerate. It was believed that pathological growth of the heart (known as cardiac hypertrophy) was due to the production of new cardiomyocytes, and that tissue damage from viral myocarditis triggered a regenerative response (reviewed in Macmahon, 1937). This idea changed when a series of detailed studies of hypertrophied hearts demonstrated that pathological cardiac growth was due to increased cardiomyocyte size without cell division (Karsner, Saphir, & Todd, 1925). Since then, multiple studies have analyzed the mammalian heart's response to different insults (Rumyantsev, 1977). In general, these experiments have shown that the adult mammalian heart does not exhibit a noticeable ability to regenerate.

Until recently, the myocardium had been considered a post‐mitotic or terminally differentiated tissue (Zak, 1973). It is generally accepted that the failure of the mammalian heart to regenerate results from the inability of adult cardiomyocytes to divide because of cell cycle exit (reviewed in Laflamme & Murry, 2011). However, this classical view has been challenged by fundamental discoveries in recent years. Seminal work by Porrello and colleagues demonstrated that the neonatal mouse heart exhibits a transient regenerative potential that disappears during the first week of postnatal life (Porrello et al., 2011, 2013). Moreover, studies using stable isotope incorporation during DNA replication have shown that a small number of cardiomyocytes are renewed during adult life in mammals (Bergmann et al., 2009, 2015; Senyo et al., 2013). Although the rate of cardiomyocyte renewal in adults is clearly insufficient to compensate for the loss of myocardium after an infarction, these results provide optimism: if the human heart has some endogenous regenerative potential, then perhaps this potential can be bolstered to promote myocardial regeneration.

### 2.1 Heart regeneration in amphibians

In contrast to the limited regenerative responses of adult mammals, other animals have remarkable capacities to regenerate. Classic work from Spallanzani on salamander limb regeneration sparked an enduring fascination with understanding how amphibians and fishes regrow injured or lost organs (Spallanzani, 1769), something that is quite evident in the numerous studies published ever since.

Some amphibians have been considered “champions of regeneration” due to their ability to repair almost any injured body part, including limbs (reviewed in Brockes & Kumar, 2008), different regions of the central nervous system (Minelli, Franceschini, Del Grande, & Ciani, 1987), the jaw (Ghosh, Thorogood, & Ferretti, 1994), parts of the intestine (O'Steen & Walker, 1962), and the retina (Keefe, 1973). Interestingly, the first evidence of vertebrate heart regeneration was obtained from studying amphibians. Ventricular injury induces cardiomyocyte DNA synthesis and karyokinesis in frogs, newts, and axolotls (Flink, 2002; Oberpriller & Oberpriller, 1971, 1974; Piatkowski, Mühlfeld, Borchardt, & Braun, 2013; Rumyantsev, 1966, 1973; Witman, Murtuza, Davis, Arner, & Morrison, 2011). Although these experiments showed that amphibians reactivate a cardiomyocyte proliferative program upon injury, most studies described incomplete restoration of the ventricular myocardium. For example, amputation of 25% of the newt ventricle results in the formation of a scar with inflammatory cells infiltrating the wound area (Oberpriller & Oberpriller, 1974). A smaller injury, generated by perforating the ventricular wall with a hypodermic needle, results in complete regeneration and reduced fibrosis (Witman et al., 2011). These differences in regenerative capacity might be explained by the severity of the injury. However, a systematic study evaluating amphibian cardiac regeneration in response to insults of different severities has yet to be performed.

Regardless of whether amphibians regenerate their hearts completely following injury, their ability to reactivate cardiomyocyte proliferation is vastly superior to that of mammals. Two pieces of evidence illustrate the higher competence of adult amphibian cardiomyocytes to reenter the cell cycle. First, at least one out of three cardiomyocytes in the adult newt heart divides efficiently in vitro (Bettencourt‐Dias, Mittnacht, & Brockes, 2003; Oberpriller, Oberpriller, Matz, & Soonpaa, 1995), a much higher percentage than that described in mammals (Engel, 2005). Second, if the amputated myocardium is minced and the mass of dissociated cardiomyocytes is transplanted back into the injured newt, the transplanted cardiomyocytes proliferate actively and the ventricle recovers more efficiently with reduced fibrosis (Bader & Oberpriller, 1978).

Although the use of amphibians in cardiac regeneration studies has declined in the last few years, probably due to the reduced availability of genetic and molecular tools, we are indebted to this pioneering work that first described the vertebrate heart's ability to regenerate.

### 2.2 The discovery of heart regeneration in zebrafish

**Figure 2:**
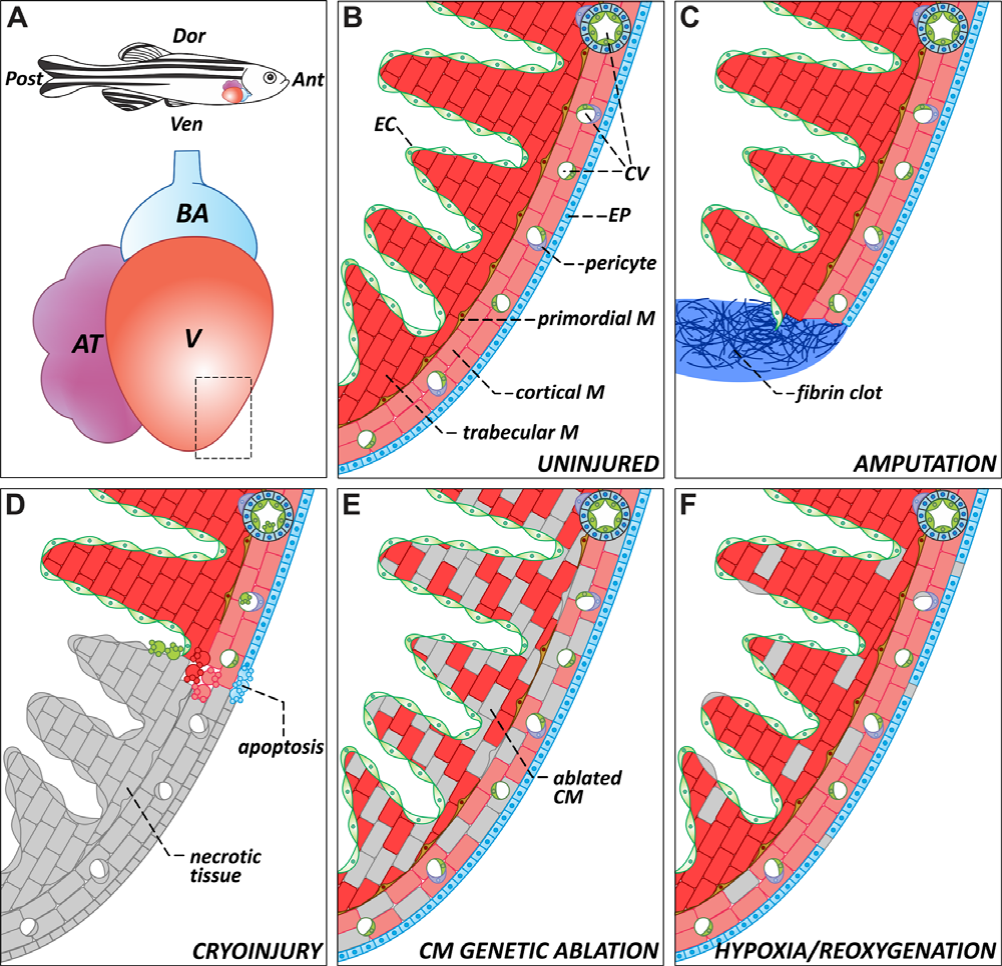
The zebrafish heart: anatomy, histology, and injury paradigms. (A) Schematic representation of the anatomical position of the heart in the adult zebrafish. The teleost heart is composed of a single atrium and a single ventricle. Blood exits the heart through the bulbus arteriosus, an elastic, non‐contractile chamber composed of smooth muscle. (B) Histological organization of the adult zebrafish ventricle. Cardiac muscle is covered externally by the epicardium and internally by the endocardium. The myocardium is divided into three distinctive populations: trabecular, primordial, and cortical. The cortical myocardium is highly irrigated by coronary vessels. Endothelial cells from the coronary vasculature are frequently surrounded by pericytes. For simplicity, the presence of fibroblasts in the uninjured heart has been omitted. (C) Apex amputation removes ∼20% of the ventricle and leads to the formation of a fibrin clot. (D) Cryoinjury induces local tissue necrosis (∼20% of the ventricle) and triggers apoptosis. (E) Cardiomyocyte genetic ablation causes diffuse loss of ∼60% of cardiomyocytes in the heart, while preserving the remaining cell types. (F) Hypoxia/reoxygenation induces low levels of diffuse cell death in all cell types of the heart. Ant, anterior; AT, atrium; BA, bulbus arteriosus; CM, cardiomyocyte; CV, coronary vasculature; Dor, dorsal; EC, endocardium; EP, epicardium; M, myocardium; Post, posterior; V, ventricle; Ven, ventral

The zebrafish (*Danio rerio*) is arguably one of the most important models for developmental and regenerative biology (reviewed in Gemberling, Bailey, Hyde, & Poss, 2013). In 25 years, hundreds of mutant strains have been identified (reviewed in Nüsslein‐Volhard, 2012), and multiple genetic tools, originally pioneered in *Drosophila* and mouse, have been successfully adapted to zebrafish. The zebrafish adult heart is simpler (has one atrium and one ventricle) and smaller (∼1 mm^3^) than the mammalian heart, but its histological composition is similar to that of other vertebrates (Fig. 2A, B). Because mutant embryos lacking active circulation are capable of surviving up to 5 days post fertilization, the zebrafish has been exceptionally exploited in developmental cardiovascular research. As an example, a large number of genes required for cardiovascular development have been identified through genetic screening strategies (reviewed in Staudt & Stainier, 2012).

Adult zebrafish have a remarkable capacity to regenerate different organs, including all seven fins (reviewed in Poss, Keating, & Nechiporuk, 2003), the retina (Vihtelic & Hyde, 2000), the spinal cord (Becker, Wullimann, Becker, Bernhardt, & Schachner, 1997), the telencephalon (Kroehne, Freudenreich, Hans, Kaslin, & Brand, 2011), and the kidney (Diep et al., 2011). However, the mechanisms that control regeneration appear to be organ‐specific. For example, fin regeneration depends on the formation of the blastema, a structure composed of dedifferentiated cells that are highly proliferative and give rise to all components of the regenerated fin (reviewed in Pfefferli & Jaźwińska, 2015). In contrast, regeneration of the telencephalon does not involve the formation of a blastema. Instead, it requires the activation of a population of resident progenitor cells characterized by the expression of the Notch target gene *her4.1* (Kroehne et al., 2011).

In 2002, Poss and Keating described the most robust cardiac regenerative response to date in a vertebrate (Poss, Wilson, & Keating, 2002). Specifically, they showed that the zebrafish heart regenerates efficiently after amputation of up to ∼20% of the ventricle. Upon resection, the heart bleeds profusely for a few seconds, but the rapid formation of a fibrin clot prevents the zebrafish from exsanguinating. The fibrin clot is replaced by new muscle in the following weeks, and an almost perfect recovery of the ventricle is achieved between 30 and 60 days post‐injury (dpi) (Poss et al., 2002; Raya et al., 2003).

Interestingly, heart regeneration is not common to all teleost species. Although hearts in other cyprinids such as the goldfish (*Carassius auratus*) and the giant danio (*Devario aequipinnatus*) regenerate successfully (Grivas et al., 2014; Lafontant et al., 2012), those in medaka (*Oryzias latipies*) scar instead (Ito et al., 2014). Exploring the differential responses of zebrafish and medaka to cardiac injury represents a unique opportunity to identify factors required for cardiac regeneration.

The pioneering discoveries from Poss and Keating opened a new field of study and raised many fascinating questions that are currently being addressed in laboratories around the world. Why does the zebrafish heart not develop a fibrotic scar? What are the cellular sources of regenerated tissue? What signals are involved in regeneration? We summarize the answers to some of these questions in the following sections.

## 3 INJURY MODELS TO STUDY HEART REGENERATION

For more than a decade, zebrafish heart regeneration was studied exclusively using ventricular resection as the injury model (Fig. 2C). In this injury paradigm, a portion of the ventricle is removed, and regeneration is scored as complete regrowth of the lost tissue (Poss et al., 2002; Raya et al., 2003). One of the more remarkable aspects of regeneration after apex amputation is the lack of scar tissue formation, which was consistent with the widely accepted idea that myocardial scarring and regeneration were mutually exclusive events (reviewed in Schulze & Lee, 2004). However, because ventricular resection is based on tissue removal rather than cell death, debris clearance is not required, which might have explained the lack of scarring. For many years, it was unknown whether the zebrafish heart was able to regenerate following more severe injuries.

More recently, a number of alternative injury models that induce tissue death have been established to study heart regeneration in the zebrafish. The reduced size of the zebrafish heart has precluded the use of common injury methods employed in larger animals such as coronary artery ligation to induce MI. The first alternative approach to resection was the cryoinjury method (Chablais, Veit, Rainer, & Jaźwińska, 2011; González‐Rosa, Martín, Peralta, Torres, & Mercader, 2011; Schnabel, Wu, Kurth, & Weidinger, 2011). In this paradigm, a metal filament is precooled in liquid nitrogen and applied to the ventricular surface to freeze a portion of it. Fast freezing and thawing of cells results in tissue necrosis followed by apoptosis of cells surrounding the necrotic area (Fig. 2D). As in human MI (Itoh et al., 1995; Saraste et al., 1997), cryoinjury results in rapid cardiomyocyte enucleation, while the sarcomeric apparatus remains relatively intact for a few days (González‐Rosa et al., 2011). Compared to apex amputation, cryoinjury induces a more severe apoptotic response that affects all cell types, including the epicardium, the endocardium, and the coronary vasculature. Shortly after injury, inflammatory cells infiltrate the damaged area, and there is extensive deposition of fibrotic tissue, concomitant with the presence of myofibroblasts at the site of the wound. In contrast to mammals, zebrafish cardiac fibrosis is transient, as the scar is efficiently cleared and repopulated by cardiomyocytes within 3−4 months. This same sequence of events occurs after heat cauterization of the heart both in giant danio and the goldfish (Grivas et al., 2014; Lafontant et al., 2012). Importantly, these results have shown that fibrosis is not inhibitory to the process of regeneration.

Ventricular regeneration after cryoinjury is significantly slower than that following other injury paradigms, even when the amount of lost tissue is similar. This delay can be explained by the need to remove necrotic material before regeneration can occur. The initial study employing cryoinjury used a copper filament to induce ventricular injury and described removal of the fibrotic scar and complete regeneration within 130 days (González‐Rosa et al., 2011). Another study reported that more severe injuries induced by a thicker copper filament regenerated after 180 dpi (Hein et al., 2015), suggesting that the severity of injury influences the recovery window. Moreover, the use of platinum instead of copper to induce cryoinjury also influences the injury size (González‐Rosa & Mercader, 2012). Cryoinjured ventricles develop signs of cardiac remodeling such as enlargement of the ventricle, thickening of the injured wall, and acquisition of a more rounded ventricular shape. Remarkably, a recent report has shown that in cryoinjured hearts the primordial layer displays incomplete regenerative capacity (Pfefferli & Jazwinska, 2017), a finding that has not been previously appreciated in regenerated hearts after apex amputation (Gupta & Poss, 2012). As we discuss later, the pumping efficiency of the heart is recovered but some areas of the ventricle remain permanently affected after cryoinjury.

A third strategy to induce cardiac injury relies on inducible genetic systems to ablate cardiomyocytes through the expression of toxins or enzymes that catalyze the production of cytotoxic metabolites. Curado and colleagues designed a transgenic system to express bacterial nitroreductase (NTR) specifically in cardiomyocytes. NTR expression alone is not toxic. However, it catalyzes the conversion of the prodrug metronidazole (Mtz) into a metabolite that induces cell death. This system offers temporal control of the ablation process because Mtz can be added to and removed from the fish water at will (Curado et al., 2007). Zhang and colleagues have used this technology to specifically ablate ventricular cardiomyocytes in the developing zebrafish heart, which uncovered the ability of atrial myocytes to acquire a ventricular phenotype during embryonic heart regeneration (Zhang et al., 2013).

To study adult regeneration, a second system has been created to conditionally express the diphtheria toxin chain A, DTA (Wang et al., 2011), in cardiomyocytes. When DTA expression is induced, it promotes diffuse ablation of up to ∼60% of all cardiomyocytes (Fig. 2E). Although this massive loss of cardiomyocytes is tolerated by the zebrafish, these animals develop signs of heart failure such as lethargy, gasping, and low tolerance to exercise. Remarkably, complete regeneration after myocardial ablation is achieved in ∼30 days with no scarring, probably because the endocardium and the epicardium (the internal and external epithelial lining of the cardiac chambers, respectively) are not affected by DTA expression. Because myocardial ablation induces cardiomyocyte death specifically, this paradigm more accurately resembles an advanced cardiomyopathy than an MI. However, it remains an excellent system to identify factors implicated in cardiomyocyte proliferation. Myocardial ablation can also be induced in a very reproducible manner in large cohorts of animals, which confers some experimental advantages. Genetic ablation systems can be used to analyze regeneration in larvae and juvenile zebrafish (Curado et al., 2007; Zhang et al., 2013), which would be ideal for performing genetic and chemical screens for regeneration phenotypes (Choi et al., 2013).

Lastly, a hypoxia/reoxygenation model has been established with the goal of better modeling human MI (Parente et al., 2013). However, because the whole animal is exposed to hypoxia, this model induces lesions that are not limited to the heart, as evidenced by inflammation in other organs. Although this treatment induces apoptosis and proliferation in the heart, it does not cause gross injuries visible by histological analysis (Fig. 2F). After hypoxia/reoxygenation, animals exhibit a transient reduction of cardiac function, but it remains to be determined whether this functional impairment is due to myocardial stunning or cardiomyocyte death. Localized hypoxia, which would more closely model human MI, has yet to be developed in zebrafish.

Overall, these studies provide abundant evidence for the exceptional regenerative ability of the zebrafish heart. In Table 1 we provide a detailed comparison of the main injury models available to researchers studying zebrafish heart regeneration. In addition to these models, which severely damage the heart, alternative strategies have been introduced to create milder injuries. These include scratching, piercing, or stabbing the adult heart (Gupta et al., 2013; Itou et al., 2014; Kikuchi, Holdway, et al., 2011). Milder insults have also been used to study regeneration during juvenile stages, when the use of other surgical procedures is extremely challenging (Gupta et al., 2013).

**Table 1:** Comparison of the approaches used to study heart regeneration in adult zebrafish

	Injury method			
	Apex resection	Cryoinjury	Genetic ablation	Hypoxia/reox
Tissue affected (%)	∼20% (ventricle)	∼25%−30% (ventricle)	60% (atrium + ventricle)	?
Tissue death (affected tissue)	– (apoptosis limited to tissue around amputation plane)	+++ (all cell types)	+++ (only cardiomyocytes)	*+*
Cardiac specific	Yes	Yes	Yes	No
Localized injury	Yes	Yes	No	No
Fibrosis	– or low	+++	–	–
Ventricular remodeling	Low	High	–	–
Hypoxia	+ (local hypoxia)	?	?	+++ (generalized hypoxia)
Functional recovery	+++ (electrical coupling, exercise tolerance)	Pumping efficiency ++ Segmental motility –	+++ (electrical coupling, exercise tolerance)	Pumping efficiency ++
Regeneration time (days)	30–60	130–80	30	NA
Requires specific transgenes?	No	No	Yes	No
Can be performed in embryonic/larval stages?	No	No	Yes	Yes

## 4 ORIGIN OF THE REGENERATED TISSUE

One of the most important tasks in the study of organ regeneration is identifying the origin of new tissue (Tanaka & Reddien, 2011). This not only is of academic interest but could also impact the design of new therapeutic strategies to regenerate damaged or lost tissue.

### 4.1 Origin of regenerated myocardium

When the first reports describing the ability of the zebrafish heart to regenerate were published (Poss et al., 2002; Raya et al., 2003), it was unknown whether cardiomyocytes within regenerated myocardium originate from a population of resident progenitor cells, from transdifferentiation of other cardiac cell types, or from proliferation of preexisting cardiomyocytes. The first experiments to address this were based on transgenic lines expressing two fluorescent proteins that fold and degrade at different rates and suggested that regenerated myocardium derives from cardiac progenitor cells (Lepilina et al., 2006). This hypothesis was supported by the reexpression of cardiac progenitor markers (*hand2*, *nkx2.5*, *tbx20*, and *tbx5*) around the injury area.

**Figure 3:**
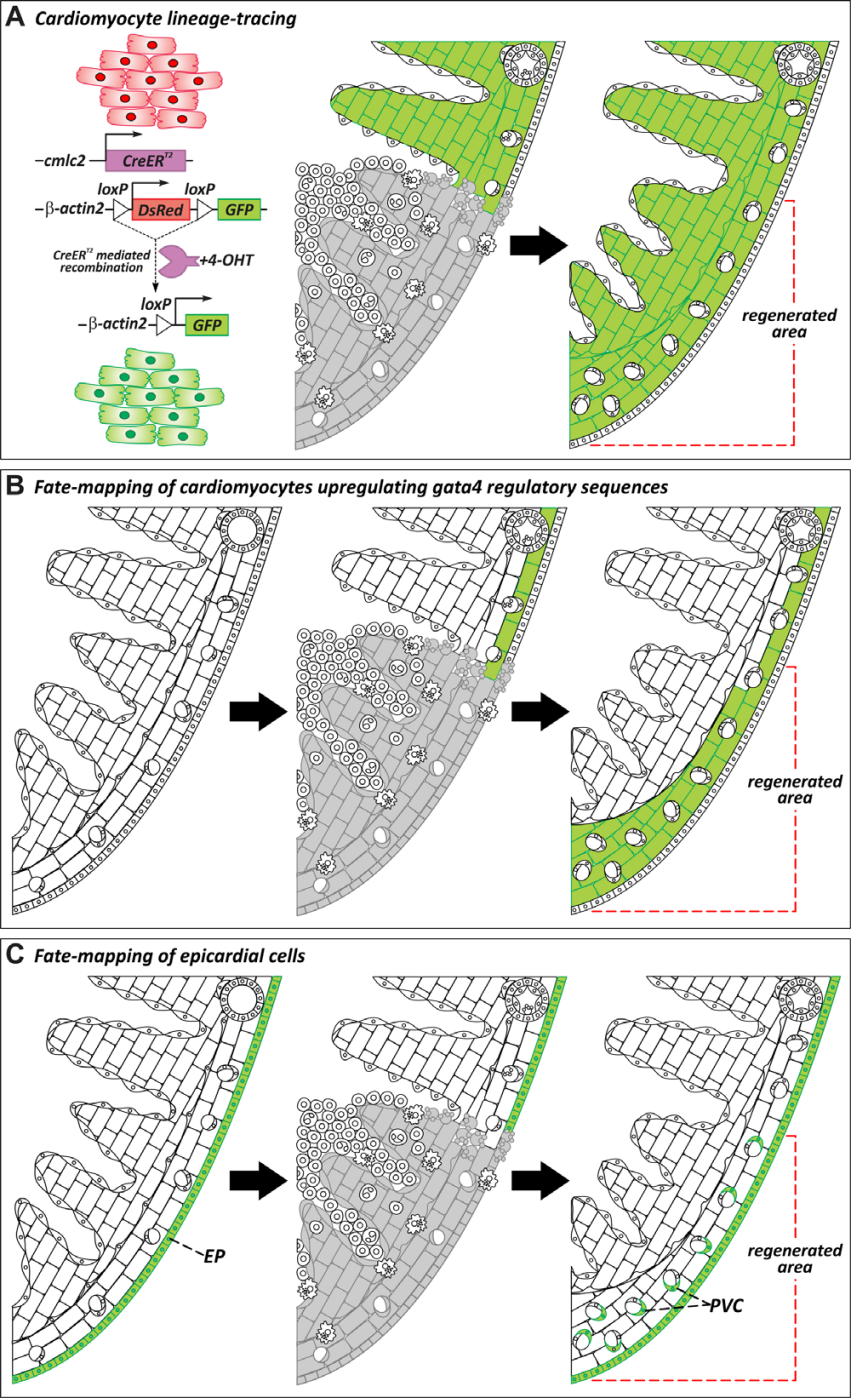
Cellular origins of regenerated tissue. (A) Genetic lineage‐tracing experiments to determine the origin of regenerated myocardium during zebrafish heart regeneration. Virtually all cardiomyocytes in uninjured hearts are labeled by GFP expression after treatment with 4‐hydroxytamoxifen (4‐OHT). In regenerated hearts, the new myocardium is GFP+, revealing that new muscle derives from the proliferation of preexisting cardiomyocytes (Jopling et al., 2010; Kikuchi et al., 2010). (B) A population of subepicardial cardiomyocytes activates regulatory sequences of *gata4* upon injury. Fate mapping of cardiomyocytes that activate *gata4* regulatory sequences reveals that these cells contribute preferentially to myocardial regeneration (Kikuchi et al., 2010). (C) Lineage tracing of epicardial cells using *tcf21:CreER^T2^* (Kikuchi, Gupta, et al. 2011) or tissue transplants (González‐Rosa et al., 2012) demonstrates that the epicardium gives rise to perivascular cells but not myocardium in the regenerated heart. EP, epicardium; PVC, perivascular cell

The origin of regenerated myocardium was subsequently revisited using Cre‐*loxP* genetic fate mapping. To perform lineage tracing of cardiomyocytes during regeneration, two independent groups generated transgenic lines expressing tamoxifen‐inducible Cre recombinase (CreER^T2^) under the regulatory sequences of the cardiomyocyte‐specific promoter *cmlc2* (*myl7*). By crossing this line to a Cre‐responsive strain, they found that cardiomyocytes in the regenerate derive from the proliferation of preexisting cardiomyocytes (Jopling et al., 2010; Kikuchi et al., 2010) (Fig. 3A). Likewise, heart regeneration in neonatal mice is also supported by reactivation of cardiomyocyte proliferation rather than by de novo differentiation of stem cells (Porrello et al., 2011), suggesting that endogenous regeneration mechanisms are evolutionarily conserved.

Following cardiac injury, proliferating cardiomyocytes in zebrafish exhibit several morphological and transcriptional changes. Similar to newt cardiomyocytes after injury (Oberpriller & Oberpriller, 1995) and mammalian cardiomyocytes during development (Ahuja, Perriard, Perriard, & Ehler, 2004), they display partial sarcomere disassembly (Jopling et al., 2010; Kikuchi et al., 2010) and acquire a partially dedifferentiated phenotype characterized by downregulation of some sarcomeric proteins and reexpression of embryonic myosins (Sallin, de Preux Charles, Duruz, Pfefferli, & Jaźwińska, 2015; Wu et al., 2016). In line with these findings, a population of subepicardial cardiomyocytes reactivates regulatory sequences of the transcription factor *gata4* after injury (Kikuchi et al., 2010). Interestingly, lineage tracing these cardiomyocytes revealed that this population contributes preferentially to myocardial regeneration (Fig. 3B). Moreover, overexpression of a dominant negative form of *gata4* in cardiomyocytes significantly impairs proliferation of cardiomyocytes from the cortical layer, suggesting that reactivation of a developmental program is required for heart regeneration (Gupta et al., 2013).

Despite these discoveries, our understanding of myocardial regeneration is still incomplete, and a number of fascinating aspects remain to be explored. What factors determine which cardiomyocytes upregulate *gata4* regulatory sequences? Are these cells “elite” cardiomyocytes and, if so, what makes them special? Is this population itself heterogeneous? Are cardiomyocytes from different myocardial compartments (trabecular, primordial, and cortical myocardium) able to contribute to other compartments during regeneration? While multicolor fate mapping of cardiomyocytes in the adult zebrafish heart suggests that cells from each myocardial compartment are restricted in their contribution during regeneration (Gupta et al., 2012), further work using compartment‐specific Cre lines is required to investigate the plasticity of adult cardiomyocytes.

### 4.2 Epicardial contribution to regeneration

The study of heart regeneration in the zebrafish has also illuminated essential roles performed by non‐muscular cells. As we discuss later, both the endocardium and epicardium are essential players during zebrafish cardiac regeneration. The original description of epicardial activation during regeneration suggested that epicardial cells revascularize the wound area (Lepilina et al., 2006). Because there are numerous pieces of evidence from mouse studies suggesting that the epicardium gives rise to a population of cardiomyocytes during development and after injury (Cai et al., 2008; Katz et al., 2012; Zhou et al., 2008), there was considerable interest in studying the contribution of the epicardium to zebrafish heart regeneration.

Two complementary approaches have been used to study the fate of epicardial cells during regeneration in zebrafish. Using genetic fate mapping of cells expressing the transcription factor *tcf21*, which labels the epicardium and a population of resident fibroblasts, Kikuchi and colleagues demonstrated that the epicardium gives rise to perivascular cells but not to endothelial cells, smooth muscle cells, or cardiomyocytes (Kikuchi, Gupta, et al. 2011). An alternative approach based on tissue transplantation demonstrated that epicardial cells infiltrate the damaged area and differentiate into myofibroblast and perivascular cells but not into cardiomyocytes or coronary endothelium (González‐Rosa, Peralta, & Mercader, 2012) (Fig. 3C). It remains to be determined whether all myofibroblasts and cells producing the transient fibrotic tissue in the zebrafish are derived from epicardial cells or whether other sources such as the endocardium or inflammatory cells are also involved.

### 4.3 Origin of the endocardium and coronary vessels

Less attention has been paid to the origins of new endocardium or the endothelial and smooth muscle cells of the regenerated coronary vasculature. Of interest, Zhao and colleagues showed that the regenerated endocardium and coronary endothelium derive from preexisting endothelial and/or endocardial cells (Zhao et al., 2014). However, these experiments did not allow for exploring whether endocardial cells give rise to coronary endothelium, as has been described during zebrafish development (Harrison et al., 2015). A more detailed analysis using endocardial‐specific Cre lines has yet to be performed.

## 5 DYNAMICS OF ZEBRAFISH HEART REGENERATION

Zebrafish heart regeneration is a very dynamic process. Much attention has been devoted to factors that stimulate myocardial proliferation during cardiac regeneration. Although we recognize that this event is crucial, we also want to emphasize the importance of events occurring shortly after injury that have a critical downstream impact on myocardial regeneration as well as interactions between cardiomyocytes and non‐cardiomyocytes. For simplicity, we have subdivided regeneration into the following chronological phases: (1) early response to injury (that includes inflammation and endocardial activation); (2) endocardial and epicardial regeneration; (3) cardiomyocyte proliferation; and (4) integration of regenerated cardiomyocytes into the myocardium and scar removal.

**Figure 4:**
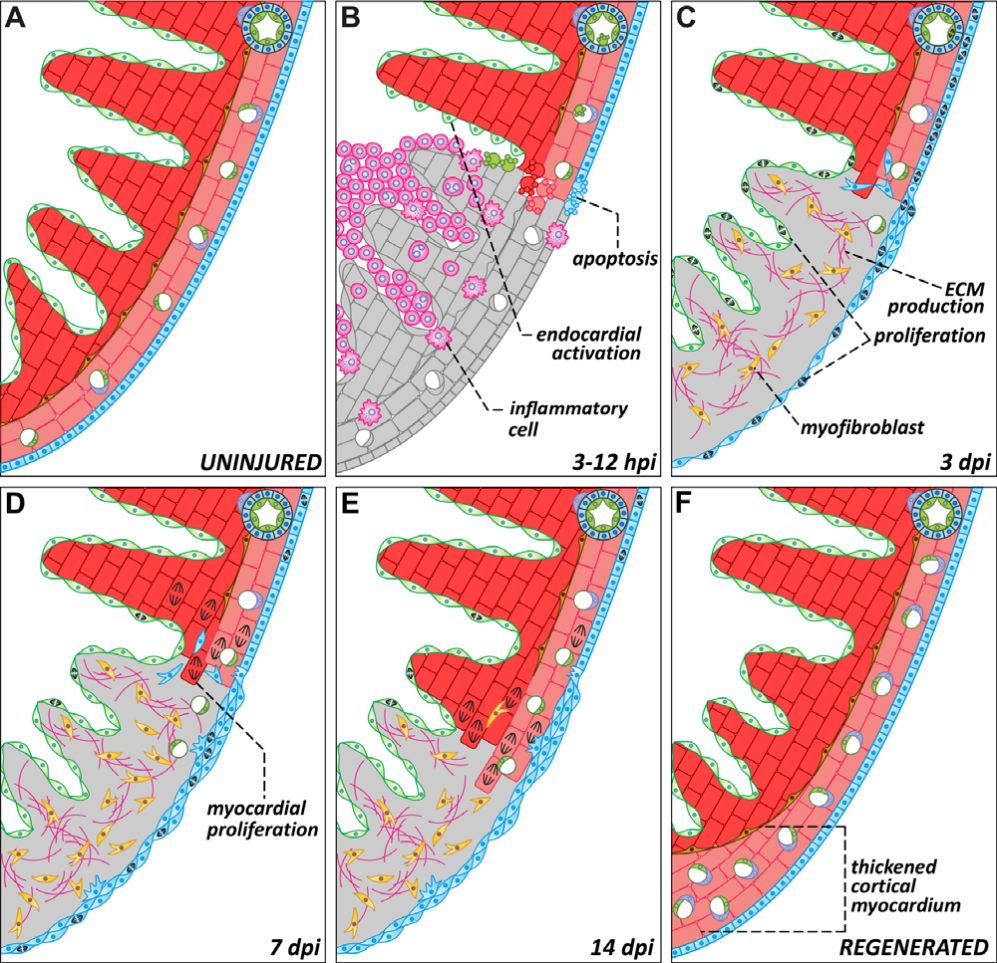
Dynamics of zebrafish heart regeneration. Representations of a region of the zebrafish heart in the absence of injury (A) or at different stages after cryoinjury (B−F). (B) Ventricular cryoinjury induces local tissue necrosis (gray) and apoptosis in all cell types around the injured region. Tissue death triggers the recruitment of inflammatory cells and endocardial activation. (C) During the first days after injury, epicardial and endocardial cells proliferate actively and cover the injured area, establishing a “regenerative scaffold.” Epicardial cells also undergo epithelial to mesenchymal transitions and invade the underlying myocardium. Myofibroblasts appear in the injury zone and there is an accumulation of extracellular matrix. (D−E) Cardiomyocytes located in the wound edge proliferate and repopulate the injured area. As the myocardium regenerates, the fibrotic tissue progressively disappears. (F) In advanced stages of regeneration, the zebrafish myocardium appears completely restored. Compared to uninjured controls or to the contralateral wall, the regenerated wall shows a significant expansion of the cortical myocardium. dpi, days post‐injury; ECM, extracellular matrix; EP, epicardium; PVC, perivascular cell

We illustrate this process in Figure 4 using the response to cryoinjury as an example, but we have also incorporated information from different injury models. Additionally, we refer the reader to Table 2, where we provide detailed information regarding signals and factors that are involved in zebrafish heart regeneration.

**Table 2:** Signaling during zebrafish heart regeneration

	**Changes induced during regeneration**	**Evidence and experimental manipulations**	**Function**	**References**
**FGF**	↑*fgf17b* (M) ↑*fgfr2, fgfr4* (EP, F)	ISH **LOF**: *hsp:dn‐fgfr1*	Promotes **epicardial EMT** and **revascularization**, facilitating muscle regeneration	Lepilina et al., 2006
**PDGF**	↑*pdgfb* (THR) ↑*pdgfrb* (EP, F)	Microarray, ISH **LOF**: PDGF chemical inhibitor	Stimulates **epicardial proliferation** and **EMT**. Required for **revascularization** and **cardiomyocyte proliferation**	Kim et al., 2010; Lien et al., 2006
**RA**	↑*raldh2* (EP, EC)	ISH **LOF**: *hsp:dn‐zrar*, *hsp:cyp26a1* **GOF**: RA and retinoid agonist	Required (but not sufficient) for **cardiomyocyte proliferation**	Kikuchi, Holdway, et al., 2011; Lepilina et al., 2006
**TGF** *β*	↑*tgfβ1*, *tgfβ2*, *tgfβ3* (EP, F, M) ↑*alk4*, *alk5a* (EP, F, EC) ↑*alk5b* (U)	ISH, IF **LOF**: TGFβ receptor and Smad3 inhibitor	Required for **ECM production** and **cardiomyocyte proliferation**.	Chablais & Jazwinska, 2012; Choi et al., 2013
**VEGF**	↑*vegfaa* (M)	qPCR **LOF**: *hsp:dn‐vegfaa; vegfaa^−/−^* (early rescued)	Required for **early revascularization** of the injured area, that later supports **cardiomyocyte proliferation**	Marín‐Juez et al., 2016
**IGF**	↑*igf2b* (EC, EP) ↑*igfr1* (M)	Microarray, ISH (*igf2b*), IF (*igfr1*) **LOF**: *hsp:dn‐igf1raGFP;* Igf‐R chemical inhibitor	Stimulates **cardiomyocyte proliferation**	Choi et al., 2013; Huang, Harrison, et al., 2013; Lien et al., 2006
**Hh**	↑*shh* (EP, SM bulbus) ↑*dhh* (SM bulbus) ↑*ptch1, gli2a* (EP) ↑*ptch2* (M)	*shh:EGFP* reporter; *ptch2:EGFP* reporter; ISH, qPCR **LOF**: Smoothened antagonist **GOF**: Shh recombinant protein	Required for **epicardial proliferation** and **directional migration**. Required for **cardiomyocyte proliferation**	Choi et al., 2013; Wang et al., 2015
**Notch**	↑*notch1a, notch2* (EP, EC) ↑*notch1b, deltaC* (EC)	ISH, qPCR, **LOF**: *hsp:dn‐maml* **GOF**: *hsp:gal4/UAS:NICD; hsp:NICD*	Correct balance in Notch signaling is required for **cardiomyocyte proliferation**, probably through paracrine signaling from endocardium and/or epicardium	Raya et al., 2003; Zhao et al., 2014
	↑*notch1b, notch2, notch3, dll4, lfng* (EC)	ISH, qPCR, RNASeq **LOF**: γ‐secretase inhibitor **GOF**: *hsp:gal4/UAS:NICD*	Both Notch inhibition and overactivation impair heart regeneration. Notch is required for **cardiomyocyte proliferation**, endocardial maturation and for restricting inflammation at the wound edge	Münch et al., 2017
**BMP**	↑*bmp2b, bmp7* (EP, EC) ↑*id2b* (EC) ↑*bmpr1aa* (WE) ↑phospho‐Smad1/5/8 (multiple cell types)	TomoSeq, ISH, IF, BMP transgenic reporter **LOF**: *hsp:nog3; bmpr1aa^−/−^*; BMP type‐I receptor antagonist. **GOF**: *hsp:bmp2b*	Required for **cardiomyocyte dedifferentiation** and **proliferation**	Wu et al., 2016
**Nrg1**	↑*nrg1* (PVC)	qPCR, RNAScope **LOF**: Erbb receptors inhibitor **GOF**: *iCM:nrg1*	Required and sufficient for **cardiomyocyte proliferation**. Triggers a regenerative program in the absence of injury.	Gemberling et al., 2015
**Cxcl12 (Sdf1)**	↑*cxcl12a* (EP) ↑*cxcr4b* (WE)	ISH, IF **LOF**: *cxcr4b^−/−^*; CXCR4 antagonist	Involved in **correct integration of cardiomyocytes in injured area**. Revascularization?	Itou et al., 2012
**Fibronectin**	↑*fn1* (EP, FB, injury specific) ↑*fn1b* (EP) ↑*itgb3* (M)	LC‐MS/MS, ISH, IF, *fn1* and *itgb3* BAC reporters **LOF**: *hsp:fn^I^1‐9 (dn‐fn)*, thermosensitive allele *fn1^TS/TS^*	Dispensable for cardiomyocyte proliferation but required for **correct integration of cardiomyocytes regenerate** and replacement of fibrotic tissue Regulation of inflammation? Epicardial migration? Revascularization?	Wang et al., 2013
**Jak/Stat**	↑*il11a* (EC) ↑*il11b, lif* (IC) ↑*il11rα, il6st, jak1, stat3, socs3b* (M)	CM‐TRAP, qPCR, ISH, WB, ChIP **LOF**: *iCM:dn‐Stat3* **GOF**: recombinant Rln3	Required for **cardiomyocyte proliferation**	Fang et al., 2013
**miRNAs**	↓*miR‐133* (M)	Microarray, ISH **LOF**: *hsp:GFP‐miR133sponge* **GOF**: *hsp:miR133*	Downregulation required for **cardiomyocyte proliferation** Among others, targets Cx43	Yin et al., 2012
	↓*miR‐100/99, let7a/7c* (M)	qPCR, ISH **LOF**: antagomir injection, Fnt antagonist **GOF**: miRNA mimic injection	Downregulation required for **cardiomyocyte proliferation** Among others, target Fntβ and Smarca5	Aguirre et al., 2014
	Early: ↓*miR‐101a* ↑*fosab* (M) Late: ↑*miR‐101a* ↓*fosab* (M)	Microarray, qPCR, RNAScope, IF **LOF**: *hsp:GFP‐miR101sponge* **GOF**: *hsp:miR101‐pre*	Early downregulation required for **cardiomyocyte proliferation** Later upregulation required for **scar removal**	Beauchemin et al., 2015
**p38** *α* **MAPK**	↓p(T180/T182)‐p38*α* (M)	IF **LOF**: *iCM:dn‐p38α* **GOF**: *iCM:caMKK6*	Downregulation required for **cardiomyocyte proliferation**	Jopling et al., 2012
**Brg1**	↑*brg1* (M, EC, EP)	ISH, IF, RNASeq, RNAScope, ChIP, Microarray, qPCR, RNAScope, IF **LOF**: *hsp:dn‐brg1; iCM: dn‐brg1; siRNA*	Required but not sufficient for **cardiomyocyte proliferation**. Interacts with Dnmt3ab to suppress expression of *cdkn1c*	Xiao et al., 2016
**Cardiogenic transcription factors**	↑*gata4* regulatory sequences (M)	*gata4:GFP* reporter **LOF**: *iCM:dn‐gata4* **GOF**: *iCM:gata4*	Required for **proliferation of cardiomyocytes in cortical layer**. Regenerating cardiomyocytes activate *gata4* regulatory sequences	Kikuchi et al., 2010 Gupta et al., 2012 Karra et al., 2015
	↑*hand2* (EC, M)	ISH, *hand2* BAC reporter **GOF**: *iCM:hand2*	Promotes **cardiomyocyte proliferation**	Lepilina et al., 2006; Schindler et al., 2014
	↑*nkx2.5* (WE) ↑*tbx5* (WE) ↑*tbx20* (WE)	ISH	Functional experiments to be performed	Lepilina et al., 2006
**Telomerase**	↑*tert* ↑telomere length	RNASeq, Telomapping **LOF**: *tert^−/−^*	Required for **cardiomyocyte proliferation**. Absence of telomerase contributes to DNA damage and senescence	Bednarek et al., 2015
**NF‐κB**	↑*nfkb1* (compact M) ↑*nfkbiaa, nfkbiab* (M)	RNASeq, qPCR, ISH, NF‐κB reporter line, ChIP. **LOF**: *iCM:IκBSR*	Required for **epicardial regeneration, cardiomyocyte dedifferentiation** and **proliferation**	Karra et al., 2015
**Inflammation**	↑*il‐1β, tnfα, il‐8, ptgs‐2b, mpx* ↑Recruitment of L‐plastin+ cells	qPCR, *lyz:GFP* reporter, IF **LOF**: chemical depletion of phagocytes and leucocytes, anti‐inflammatory drugs.	Required for **transient scar deposition**, revascularization and **cardiomyocyte proliferation**	Huang, Yang, et al., 2013; de Preux Charles et al., 2016
**MMPs**	↑*mmp2, mmp14a* (7‐30 dpi) ↑*mmp9, mmp13a/b* (7‐14 dpi)	qPCR, ISH, *in situ* zymography assay	Functional experiments to be performed	Gamba et al., 2017
**ROS**	↑*duox, nox2* (EP)	RNA‐Seq, ISH, transgenic reporter, chemical redox reporters. **LOF**: *cmlc2:Catalase‐DsRed*, chemical inhibitors	Required but not sufficient for **cardiomyocyte proliferation** (via degradation of Dusp6) Required for **revascularization**	Han et al., 2014
**Hypoxia**	↑Hypoxyprobe staining (WE)	Hypoxiprobe **LOF**: *iCM:dn‐hif1a* **GOF**: anemia induction	Required for **cardiomyocyte proliferation**	Jopling et al., 2012

CM‐TRAP, cardiomyocyte‐specific translating ribosome affinity purification; ChIP, chromatin immunoprecipitation; EC, endocardium; ECM, extracellular matrix; EP, epicardium/EPDCs; F, fibroblasts; FB, fibroblasts; GOF, gain of function; hsp, heat‐shock inducible transgenic; IC, inflammatory cells; iCM: inducible conditional expression of a given transgene in cardiomyocytes; IF, immunofluorescence; ISH, in situ hybridization; LC‐MS/MS, liquid chromatography tandem mass spectrometry; LOF, loss of function; M, myocardium; PVC, perivascular cells; qPCR, quantitiative reverse transcription polymerase chain reaction; SM, smooth muscle; THR, thrombocytes; TS, thermosensitive mutant; U, ubiquitous; WB, western blot; WE, wound edge.

### 5.1 Early response to cardiac injury: inflammation and endocardial activation

Regardless of the method used to generate lesions, injury to the zebrafish heart triggers an early response that includes inflammation (Fig. 4A, B). Expression of proinflammatory cytokines and molecules mediating the recruitment of phagocytes and neutrophils is detectable as early as 3 h post‐injury (hpi) (Huang, Yang, et al., 2013). Although our understanding of the role of inflammation in regeneration is still limited, several lines of evidence suggest that this early inflammatory response is essential for regeneration. Treatment with anti‐inflammatory drugs or chemical depletion of phagocytes and neutrophils impair revascularization and cardiomyocyte proliferation, leading to the accumulation of a fibrotic scar (Huang, Yang, et al., 2013; de Preux Charles, Bise, Baier, Marro, & Jaźwińska, 2016). These results are in agreement with findings from zebrafish brain regeneration, where inflammation is both necessary and sufficient to activate injury‐induced programs, including proliferation and neurogenesis (Kyritsis et al., 2012). It is still unknown whether a specific class of immune cells has pro‐regenerative properties and what molecular pathways are activated by these cells to promote tissue recovery.

Concomitant with the early inflammatory response, the endocardium reacts to injury in an organ‐wide manner. In uninjured hearts, endocardial cells are flat and tightly attached to the adjacent myocardium. Within hours of injury, endocardial cells respond by becoming rounded and partially detaching from cardiomyocytes (Kikuchi, Holdway, et al., 2011). These morphological changes are accompanied by reexpression of embryonic markers, such as the retinoic acid (RA) synthesizing enzyme *raldh2* (*aldh1a2*) and the transmembrane receptor *heart of glass* (*heg*). Initially, these changes affect all endocardial cells, but the alterations become quickly restricted to the injured area. Although it is still unknown what signals induce and restrict *raldh2* expression, some experimental evidence points to a role for inflammation (Kikuchi, Holdway, et al., 2011). As we discuss below, RA signaling from the endocardium is required for cardiomyocyte proliferation and myocardial regeneration.

Both the activated endocardium and inflammatory cells release cytokines (such as *il11a* and *il11b*) that instruct cardiomyocytes to proliferate. These secreted factors activate the Jak1/Stat3 axis in cardiomyocytes, which results in upregulation of *relaxin3a*, a secreted factor that stimulates cardiomyocyte proliferation (Fang et al., 2013). Cardiomyocyte‐specific expression of a dominant negative form of *stat3* during regeneration drastically reduces cardiomyocyte proliferation and blocks regeneration. These results illustrate the essential role of early inflammation on regeneration.

Another early response to injury that has remained unappreciated until recently is the rapid recovery of the coronary vasculature. Using cryoinjury, Marín‐Juez and colleagues showed that revascularization is initiated at 15 hpi, with vascular sprouting observed in the wound region. Interestingly, inhibition of early revascularization through overexpression of a dominant negative form of *vegfaa* reduces cardiomyocyte proliferation and permanently blocks regeneration (Marín‐Juez et al., 2016). In agreement with these findings, *cxcr4a* ^−/−^ adult animals that lack a well‐established vascular network are unable to regenerate completely (Harrison et al., 2015). Taken together, these results highlight the importance of the early and robust revascularization of the injured area during cardiac regeneration.

### 5.2 Endocardial and epicardial regeneration: establishment of a “regenerative scaffold”

The recovery of both the epicardium and the endocardium is achieved in the first days after injury and precedes myocardial regeneration. These non‐muscular cells play an essential role in providing an environment that facilitates myocardial proliferation.

Following activation, the endocardium proliferates actively and quickly regenerates to provide an internal covering for the wound. Münch and colleagues have recently studied endocardial recovery in detail following cryoinjury. They demonstrate that endocardial cells surrounding the wound robustly proliferate between 3 and 5 dpi, prior to the peak of cardiomyocyte proliferation at 7 dpi (Bednarek et al., 2015; Münch, Grivas, Gonzalez‐Rajal, Torregrosa‐Carrión, & la Pompa, 2017). Thereafter, endocardial cells acquire a motile phenotype and migrate to cover the wound area, after which they reorganize into a coherent epithelium. Endocardial maturation is Notch‐dependent, as inhibition of Notch signaling reduces the percentage of mature endocardial cells in the injured area. Notch activation is also required for resolving early inflammation (Münch et al., 2017).

The epicardium also becomes activated and reexpresses embryonic genes such as *raldh2*, *tbx18*, and *wt1b* upon injury (González‐Rosa et al., 2011; Lepilina et al., 2006). Similar to the endocardium, epicardial activation is initially organ‐wide but becomes restricted to the wound region within days of insult. Epicardial cells become highly proliferative and invade the underlying tissue, giving rise to epicardial‐derived cells (EPDCs) in a process resembling the epithelial to mesenchymal transition (EMT) (Lepilina et al., 2006) (Fig. 4C). Cells from the epicardium experience morphological changes as early as 12 hpi when they detach from neighboring cells and lose their characteristic epithelial shape (González‐Rosa et al., 2012). Fgf and Pdgf are secreted by cardiomyocytes and thrombocytes, respectively, to induce epicardial EMT and EPDC mobilization. Inhibition of these pathways results in defective revascularization, reduced numbers of myofibroblasts, and impaired regeneration (Kim et al., 2010; Lepilina et al., 2006).

Shortly after injury, the damaged area is covered by regenerated epicardium. Recently, Wang and colleagues have elegantly demonstrated that epicardial migration is directional, from the base of the heart to the apex. Epicardial migration over the wounds is essential, as genetic ablation of *tcf21*+ cells significantly reduces cardiomyocyte proliferation and impairs regeneration. Combining this approach with an ex vivo culture protocol to image epicardial regeneration, the authors discovered that Hedgehog signaling is essential for controlling epicardial migration. Interestingly, they found that Hedgehog ligands are produced by smooth muscle cells from the bulbus arteriosus, further illustrating the complexity of tissue interactions during cardiac regeneration (Wang, Cao, Dickson, & Poss, 2015).

Both the epicardium and the endocardium create a “regenerative scaffold” that provides support and guidance during myocardial regeneration. Specifically, the epicardium and EPDCs strongly express extracellular matrix proteins such as fibronectin, periostin, collagen I and collagen XII (González‐Rosa et al., 2012; Marro, Pfefferli, de Preux Charles, Bise, & Jaźwińska, 2016; Wang et al., 2013). Epicardial production of Cxcl12 is also involved in guiding cardiomyocytes into the wound area and chemical inhibition of Cxcr4 receptors or prevention of fibronectin production significantly affects the correct integration of cardiomyocytes into the regenerated area (Itou et al., 2012; Wang et al., 2013). Recently, Chen and colleagues have explored the therapeutic potential of this “regenerative scaffold.” Remarkably, injection of purified extracellular matrix from regenerating zebrafish hearts into the infarcted mouse heart induced cardiomyocyte proliferation and improved function (Chen et al., 2016). Further characterization of the beneficial effects of the zebrafish extracellular matrix might inform the design of pro‐regenerative biomaterials.

### 5.3 Cardiomyocyte proliferation

Wound edge cardiomyocytes receive signals from non‐myocardial cells that induce their proliferation (Fig. 4D). Among other signals, cardiomyocytes are exposed to Pdgf, RA, Igf, Shh, Tgfβ ligands, BMP and Nrg1 (Chablais & Jaźwińska, 2012; Choi et al., 2013; Gemberling, Karra, Dickson, & Poss, 2015; Huang, Harrison et al., 2013; Kikuchi, Holdway, et al., 2011; Kim et al., 2010; Wu et al., 2016). These signals are secreted from the epicardium, EPDCs, the endocardium, and circulating cells (Table 2). NF‐κB activity is also induced in cardiomyocytes during regeneration, probably by inflammatory cytokines released from circulating cells. Suppression of NF‐κB signaling blocked cardiomyocyte dedifferentiation and proliferation, and impaired epicardial regeneration (Karra, Knecht, Kikuchi, & Poss, 2015). Lastly, Notch signaling is required for myocardial proliferation and probably regulates several processes during heart regeneration (Münch et al., 2017; Zhao et al., 2014). Münch and colleagues recently reported that upregulation of Notch in endocardial cells correlates with downregulation of *serpine1*, and that chemical inhibition of *serpine1* induces endocardial and myocardial proliferation (Münch et al., 2017).

Cardiomyocyte proliferation is not only stimulated by epicardial and endocardial cells. The pro‐regenerative role of nerves during zebrafish heart regeneration has been described recently (Mahmoud et al., 2015). Nerves are well known to be essential for regeneration of other body parts, such as salamander limbs and tadpole tails (reviewed in Kumar & Brockes, 2012). In zebrafish, nerves are present on the surface of the ventricle, and they regrow into the regenerated area upon injury. Prevention of cardiac innervation through cardiomyocyte‐specific overexpression of the neural chemorepellent *semaphorin3aa* reduces cardiomyocyte proliferation and impairs myocardial wall regeneration. Consistently, chemical inhibition of cholinergic but not adrenergic signaling impairs myocardial regeneration. Interestingly, this pro‐regenerative role of nerves is conserved in mouse neonates. A detailed characterization of this phenomenon revealed that nerve production of Nrg1, but not acetylcholine, stimulates cardiomyocyte proliferation following injury of the neonatal mouse heart (Mahmoud et al., 2015). In zebrafish Nrg1 is necessary and sufficient to induce cardiomyocyte proliferation, but its source is perivascular cells rather than nerves (Gemberling et al., 2015).

Environmental and systemic factors such as oxygen tension and stress induced by animal crowding also impact regeneration (Jopling, Suñe, Faucherre, Fabregat, & Izpisua Belmonte, 2012; Sallin & Jaźwińska, 2016). Interestingly, the wound becomes hypoxic shortly after ventricular resection, and low oxygen tension is necessary for cardiomyocytes to proliferate. Experimental hyperoxia blocks regeneration, while induction of hypoxia stimulates cardiomyocyte proliferation (Jopling et al., 2012). Remarkably, fate mapping of hypoxic cardiomyocytes in mice identifies a population of cells that retain neonatal characteristics and contribute to myocardial turnover (Kimura et al., 2015), suggesting that hypoxia plays a conserved role in cardiomyocyte proliferation.

So far we have discussed the paracrine signals that induce cardiomyocyte proliferation, but what are the cardiomyocyte‐autonomous responses elicited by injury? As mentioned previously, regenerating cardiomyocytes located around the wound edge exhibit several morphological and transcriptional changes. The latter are mediated, at least in part, by epigenetic mechanisms. Xiao and colleagues recently described the role of chromatin‐remodeling factor Brg1 (Smarca4) in zebrafish heart regeneration. Brg1 expression is strongly induced upon injury in the wound edge, and overexpression of a dominant negative Brg1 blocks cardiomyocyte proliferation. The authors showed that Brg1 is required to prevent expression of cyclin‐dependent kinase inhibitors by increasing DNA methylation in collaboration with the methyltransferase Dnmt3ab (Xiao et al., 2016). It remains to be explored whether additional epigenetic mechanisms are involved in reactivating cardiomyocyte proliferation.

miRNAs also play a role in the modulation of gene expression during zebrafish heart regeneration (Aguirre et al., 2014; Beauchemin, Smith, & Yin, 2015; Yin, Lepilina, Smith, & Poss, 2012). To date, several miRNAs that control expression of genes involved in cell cycle progression have been identified. Interestingly, changes triggered in response to injury in zebrafish are not conserved in mammals, suggesting that miRNA expression contributes to the high regenerative capacity of the zebrafish heart (Aguirre et al., 2014; Crippa et al., 2016). While miRNAs represent an attractive therapeutic target, we must first understand when and where specific miRNAs are required to facilitate regeneration. For example, Beauchemin and colleagues recently reported that miR‐101a downregulation is essential for cardiomyocyte proliferation in the first days upon resection. Interestingly, they also demonstrated that miR‐101a upregulation is later required to promote fibrotic tissue removal (Beauchemin et al., 2015). These results illustrate how the dynamic regulation of miRNA expression appears to be essential for different regenerative phases.

Although a number of factors have already been identified, uncovering other pathways that underlie injury‐induced cardiomyocyte proliferation is essential for deciphering the molecular mechanisms governing zebrafish heart regeneration. Several strategies have been used to characterize this phenomenon, including transcriptional profiling and mass spectrometry of regenerating hearts (Lien, Schebesta, Makino, Weber, & Keating, 2006; Sleep et al., 2010; Wang et al., 2013). Recently, Wu and colleagues used TomoSeq to identify genes expressed in different regions of the cryoinjured heart (Wu et al., 2016). Using this strategy, they identified several transcripts upregulated specifically in the injured area, the border zone, and the uninjured myocardium.

A few studies have focused on identifying lineage‐specific changes during regeneration. Fang and colleagues performed translating ribosome affinity purification (TRAP) to identify transcriptional changes in cardiomyocytes. To that end, they generated a transgenic line that drives the expression of the large subunit ribosomal protein L10a fused to green fluorescent protein (GFP) specifically in cardiomyocytes. This strategy allowed them to isolate actively translated mRNAs from regenerating myocardium (Fang et al., 2013). Furthermore, Cao and colleagues have studied the transcriptional profile of the epicardium and fibroblasts, using single‐cell RNA sequencing of sorted *tcf21:nucGFP+* cells. Among other genes, they found that *caveolin1*, an essential molecule for exocytosis, is expressed in epicardial and fibroblast cells. Interestingly, *caveolin1* mutant animals develop normally but fail to regenerate as efficiently as wild‐type siblings. The mechanism by which caveolin1 contributes to regeneration remains obscure, as epicardial migration, revascularization, fibrin deposition, and cardiomyocyte proliferation appear unaffected in mutant animals (Cao et al., 2016).

While the number of studies that use proteomic and transcriptomic approaches to profile heart regeneration increases steadily, the number of genes potentially involved in regeneration multiplies exponentially. Nonetheless, functional validation of identified genes is slow. Recently, dynamic co‐expression network analyses have emerged as a way to identify potential candidates involved in heart regeneration (Rodius et al., 2014, 2016). This in silico approach integrates information from mammalian studies and provides online tools to visualize gene expression data. These strategies could potentially help researchers to select the most promising candidates for more intensive investigation.

### 5.4 Reintegration of cardiomyocytes into the myocardium and scar removal

The process of scar removal and reintegration of regenerated cardiomyocytes is the least explored of all phases of zebrafish heart regeneration. The extracellular matrix that is deposited shortly after injury is gradually replaced by regenerated myocardium during the weeks that follow a ventricular insult (González‐Rosa et al., 2011). Activated myofibroblasts also disappear progressively, but it is still unknown whether they die or remain as inactivated fibroblasts in the regenerated area. What cell type is responsible for clearing the collagenous scar? Are macrophages or other inflammatory cells involved? Is the composition of this transient fibrotic tissue different from the irreversible scarring that is produced after an MI in mammals?

To gain insight into scar resolution, Gamba and colleagues recently characterized the collagenolytic activity induced after cryoinjury. They detected collagen degradation in the wound region at 14 and 30 dpi, overlapping with areas of active collagen deposition. This increase in collagenolytic activity coincides with the increased expression of several matrix metalloproteinases. Specifically, they found that *mmp2* and *mmp14a* expression is significantly upregulated at 14 and 30 dpi, concomitant with the onset of scar clearance (Gamba, Amin‐Javaheri, Kim, Warburton, & Lien, 2017). It remains to be determined whether the same cells that synthesize collagen are also responsible for matrix degradation.

## 6 *IN FUNCTION VERITAS*: FUNCTIONAL EVALUATION OF REGENERATED MYOCARDIUM

Because regeneration refers to the recovery of both structure and function of an injured organ, a key goal is to determine whether the regenerated myocardium is electrically coupled and works synchronously with the rest of the ventricle.

In the first regeneration study, visual inspection was used to determine that ventricular contraction was grossly normal in regenerated hearts (Poss et al., 2002). Years later, optical mapping was used to determine that regenerated myocardium becomes electrically coupled by 30 dpi (Kikuchi et al., 2010). However, this technique prevents longitudinal studies throughout the regenerative window because the heart must be dissected from the animal.

A number of studies have utilized electrocardiograms as a non‐invasive method to examine the electrical activity of the heart after both resection and cryoinjury (Chablais et al., 2011; Yu, Li, Parks, Takabe, & Hsiai, 2010). These studies have demonstrated that injury causes prolongation of the QT segment, which is the time from depolarization to repolarization of the ventricle. As regeneration proceeds, the QT interval returns to normal, demonstrating that regeneration corrects the electrical abnormalities induced by injury.

Cardiac performance has been measured indirectly in the zebrafish using exercise tolerance tests. These experiments measure the ability of animals to swim against water flowing at different velocities. Following genetic ablation of ∼60% of cardiomyocytes, animals initially demonstrate a low tolerance to exercise that eventually improves at more advanced stages of regeneration. By contrast, animals subjected to amputation of ∼20% of the ventricle do not exhibit changes in exercise tolerance (Wang et al., 2011). Therefore, while this test does not require expensive instruments to assess cardiac function, its relatively low sensitivity means that only the most severe functional deficits will be identified.

In mammals, the standard non‐invasive technique to evaluate cardiac function is echocardiography. Recently, several groups have adapted this technology to measuring heart function in zebrafish (González‐Rosa et al., 2014; Hein et al., 2015; Huang et al., 2015; Sun, Lien, Xu, & Shung, 2008). These studies have shown that, although the pumping efficiency of the heart is recovered following cryoinjury, regenerated myocardial regions display abnormalities in wall movement (González‐Rosa et al., 2014; Hein et al., 2015). It remains unknown whether localized defects also develop after apical resection. Echocardiography was used to demonstrate that Nrg1‐induced cardiac hyperplasia eventually undermines cardiac function (Gemberling et al., 2015).

The major limitations of echocardiography are the requirement of specialized instruments and, until recently, the lack of standardized protocols for acquisition and analysis. In a recent study, Wang and colleagues performed a systematic comparison of different factors that may contribute to the variability in echocardiographic measurements in zebrafish, including anesthesia used, gender, and age. The authors also provide useful guidelines for data acquisition and validate their protocol in animals after genetic ablation of cardiomyocytes and in animals experiencing chronic anemia (Wang et al., 2017). Overall, the use of such standardized protocols will provide invaluable information to assess the degree of functional recovery in zebrafish cardiac regeneration studies.

## 7 CURRENT LIMITATIONS AND FUTURE PERSPECTIVES

Despite the tremendous amount of information obtained using the zebrafish as a model to understand heart regeneration, there are some significant limitations that should be considered.

First, some of the experimental approaches currently used to study zebrafish heart regeneration lack tissue specificity. For example, the requirement of a certain gene or pathway during regeneration is frequently evaluated using heat‐shock inducible lines (Table 2). Although this system is useful and informative, it is important to remember that a constitutively active or dominant negative form of a given protein is expressed in multiple cell types. To refine these experimental findings, investigators must identify the relevant lineage(s) in which the protein is functioning to cause the observed phenotypes. This can be accomplished using conditionally activatable transgenes and tissue‐specific Cre lines (Karra et al., 2015). Alternatively, gRNA‐guided activation of endogenous genes using tissue‐specific expression of catalytically inactive Cas9 (dCas9) fusion proteins might be an elegant and sophisticated option to zebrafish researchers in the future (reviewed in Shalem, Sanjana, & Zhang, 2015).

Second, most loss of function studies are currently performed using overexpression of dominant negative proteins, rather than conditional genetic deletions or tissue‐specific genome editing (Table 2), both of which afford greater specificity. Recently, precise genome editing has become a reality in zebrafish with the CRISPR−Cas9 system (Hwang et al., 2013), and the application of this technology has already begun to benefit the zebrafish community (Ablain, Durand, Yang, Zhou, & Zon, 2015; Hoshijima, Jurynec, & Grunwald, 2016).

Despite these limitations, compelling findings from zebrafish have inspired scientists to revisit the mammalian heart's response to myocardial injury. For instance, similarities between the hearts of neonatal mice and zebrafish provided the rationale to test for cardiac regeneration in mouse pups (Porrello et al., 2011). While mouse neonatal cardiomyocytes are morphologically equivalent to those of adult zebrafish, loss of regenerative capacity coincides with myocardial maturation within the first week of life (Vivien, Hudson, & Porrello, 2016). It has been proposed that several changes that happen during maturation – metabolic adaptations, exposure to reactive oxygen species, or cardiomyocyte polyploidization – might be responsible for this loss of regenerative potential (Vivien et al., 2016). However, these hypotheses remain to be experimentally tested. Inducing each of these adaptations experimentally in the highly regenerative zebrafish heart could reveal their individual contributions to inhibiting cardiac regenerative capacity.

Although pathways that induce cardiomyocyte proliferation have been identified, their activation will need to be directed specifically to cells in and around the infarcted area. Recently, Kang and colleagues described a *leptin b* enhancer (*LEN*) that is activated during fin and heart regeneration in zebrafish (Kang et al., 2016). Interestingly, this enhancer can be used to direct the expression of pro/anti‐regenerative factors and therefore affect regeneration in zebrafish. Although the *LEN* sequence is not highly conserved in mammals, injury to neonatal mice activates *LEN*‐lacZ transgenes. The use of such injury‐induced enhancers could allow for efforts to direct the expression of pro‐regenerative factors identified in zebrafish to promote heart regeneration in mammals.

What have we learned in 15 years about zebrafish heart regeneration? There is a solid amount of work demonstrating that adult zebrafish efficiently regenerate their hearts following several different forms of injury. Myocardial regeneration in zebrafish is not based on stem cells or transdifferentiation of other cell types but on the proliferation of preexisting cardiomyocytes. The endocardium, epicardium, and inflammatory cells play indispensable supporting roles in this process. Despite many important advances in the past 15 years, our understanding of zebrafish heart regeneration remains incomplete. There are a number of open questions that remain to be explored for each phase of regeneration. What are the first signals that trigger regeneration? Is there a unique population of cardiomyocytes that is able to respond to injury? If so, what characterizes them? What are the signals that determine when regeneration is complete? What is the role of metabolism in regeneration? Do cell‐specific properties, such as ploidy, determine the regenerative potential of a cardiomyocyte? How is the fibrotic tissue cleared? What epigenetic changes are involved in regulating regeneration? How can we apply this knowledge to the treatment of patients who suffer an MI? Deciphering the cellular and molecular mechanisms that regulate cardiac regeneration in the zebrafish will inform the development of therapeutic strategies to treat heart disease, impacting millions of people each year.
